# Low-Cost CO Sensor Calibration Using One Dimensional Convolutional Neural Network

**DOI:** 10.3390/s23020854

**Published:** 2023-01-11

**Authors:** Sharafat Ali, Fakhrul Alam, Khalid Mahmood Arif, Johan Potgieter

**Affiliations:** 1 Department of Mechanical and Electrical Engineering, Massey University, Auckland 0632, New Zealand; 2 Massey Agrifood Digital Lab., Massey University, Palmerston North 4410, New Zealand

**Keywords:** 1DCNN, air quality monitoring, calibration, low-cost CO sensor

## Abstract

The advent of cost-effective sensors and the rise of the Internet of Things (IoT) presents the opportunity to monitor urban pollution at a high spatio-temporal resolution. However, these sensors suffer from poor accuracy that can be improved through calibration. In this paper, we propose to use One Dimensional Convolutional Neural Network (1DCNN) based calibration for low-cost carbon monoxide sensors and benchmark its performance against several Machine Learning (ML) based calibration techniques. We make use of three large data sets collected by research groups around the world from field-deployed low-cost sensors co-located with accurate reference sensors. Our investigation shows that 1DCNN performs consistently across all datasets. Gradient boosting regression, another ML technique that has not been widely explored for gas sensor calibration, also performs reasonably well. For all datasets, the introduction of temperature and relative humidity data improves the calibration accuracy. Cross-sensitivity to other pollutants can be exploited to improve the accuracy further. This suggests that low-cost sensors should be deployed as a suite or an array to measure covariate factors.

## 1. Introduction

Urban air pollution has been linked to adverse effects on the environment, public health and quality of life [1]. Therefore, there is a concerted effort to alleviate the effects of air pollution [2]. Monitoring air pollution can raise awareness among the general public and subsequently lead to a sustainable urban environment [3]. Conventional air quality monitoring system typically involves the deployment of a small number of expensive stationary stations [4]. While the data from these stations are accurate, the poor spatial resolution hinders the generation of robust, city-wide air quality data. Low-cost sensors have been identified as an option to supplement the information captured by conventional air quality monitoring systems [4,5]. Many countries around the world [6,7,8] have started to adopt this approach to monitor urban pollutant at high spatial resolution.

Researchers have developed many low-cost sensors in the past few decades to capture real-time air pollution data [9,10]. Two of the most widely reported low-cost gas sensors to measure ambient air pollution are Metal Oxide (MOX) [11] and Electrochemical (EC) [12] gas sensors. Such sensors have been used in various scenarios such as road-side pollution measurement [7,13], rural and urban air pollution measurement [8,14,15,16,17,18], mobile vehicular pollution measurement [19], source attribution [20], personal exposure monitoring [4], etc. However, the data generated by these sensors are not as accurate as the measurements produced by the conventional sensors [6,21,22] due to the influence of many covariate factors. For example, EC sensors developed for measuring common pollutants, such as Carbon Monoxide (CO), Nitrogen Dioxide (NO_2_), and Ozone (O_3_) are impacted by ambient temperature, relative humidity, and cross-sensitivity to other gases [12,13,17,21,23,24]. Researchers have been working to develop calibration strategies and techniques to improve the accuracy of low-cost sensors. Such sensors can be calibrated by co-locating them with accurate sensors so that the calibrated measurements of the low-cost sensor closely agree with the co-located accurate reference sensor [9]. The co-located measurements are often performed during “field deployment” as it is difficult to emulate the inherently complex nature of the ambient conditions in a controlled lab setup [8,14,25,26]. The key component of the calibration is the training of regression models to capture the complex, often nonlinear, relationship between the raw sensor output and the ground truth provided by the accurate reference sensor.

Many computational calibration approaches to improve the accuracy of low-cost sensors have been reported in the literature. Classic statistical regressions such as Multiple Linear Regression (MLR) are still being employed in recent works [6,27,28,29,30,31,32,33]. State-of-the-art calibration methods include supervised Machine Learning (ML) techniques such as Support Vector Regression (SVR) [34,35,36,37,38,39], ensemble ML techniques, such as Random Forest Regression (RFR) [8,34,36,40,41,42,43], and Neural Networks (NN) such as Multilayer Perceptron (MLP) [25,27,28,37,38,39,43,44]) and Recurrent Neural Networks (RNN) [37,38,39,40,45,46]. Table 1 summarizes the ML techniques used for the calibration of low-cost gas sensors. With a few exceptions [6,37], most of these studies typically utilize one set of data to demonstrate the calibration performance, making it difficult to ascertain the generalizability of the techniques.

Our literature review shows that among the NN-based techniques, One Dimensional Convolutional Neural Network (1DCNN) has not been well investigated for low-cost gas sensor calibration. 1DCNN has demonstrated excellent performance for a variety of applications (e.g., indoor localization [47], human activity recognition [48], and time series forecasting [49]). However, there are only two reports [50,51] of 1DCNN being utilized for the calibration of air pollution monitoring. Kureshi et al. [50] employed it for the calibration of Particulate Matter (PM) sensors. In a recent publication that investigated the impact of the pandemic on air quality, Vajs et al. [51] employed 1DCNN to calibrate low-cost NO_2_ sensors. However, they did not benchmark its performance against any other ML techniques; therefore, it is impossible to ascertain its (comparative) efficacy. Similarly, the Gradient Boosting Regression (GBR), an ensemble learning technique, has also not been widely utilized for gas sensor calibration, although it has shown good performance in other applications (e.g., PM sensor calibration [52] and prediction and forecasting [53,54]). Bagkis et al. [41] report the only work that employed GBR for gas sensor calibration. However, their work mainly focused on temporal drift correction, and the performance of GBR was not benchmarked against sophisticated techniques such as NNs.

Contribution Statement:

- This paper proposes applying 1DCNN and GBR for calibrating low-cost CO sensors. As far as we know, this is the first work that benchmarked these algorithms against NN-based algorithms.
- Furthermore, this work, in contrast to most studies reported in the literature, evaluates the calibration models across multiple datasets, enabling us to draw more robust conclusions.
- We show that 1DCNN-based calibration is consistently accurate compared to several Machine Learning (ML) based techniques across three large CO datasets.
- We also highlight that GBR, an ML technique that has not been investigated widely for low-cost gas sensor calibration, performs quite accurately for all three datasets.

## 2. Dataset Description

**Figure 1:**
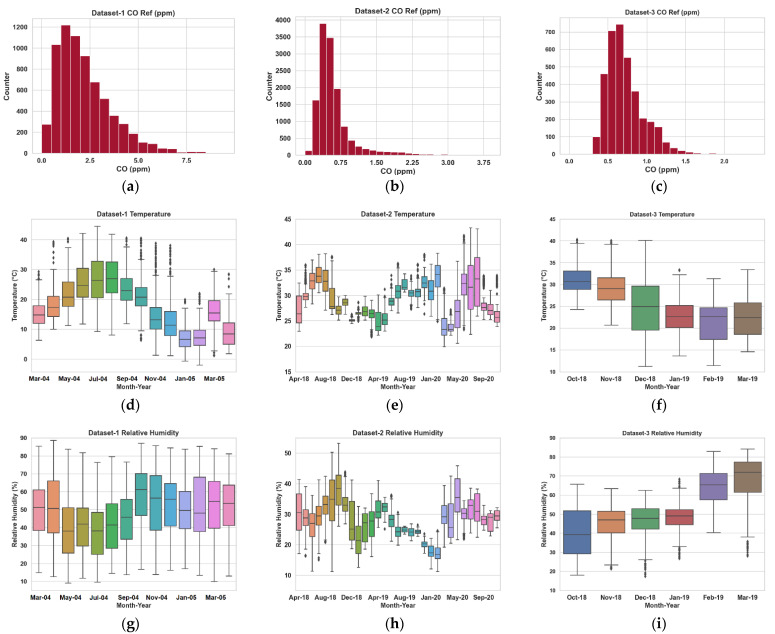
Distribution (histogram) of reference CO data in ppm for datasets 1, 2, and 3 in (**a**), (**b**) and (**c**), respectively. The median and standard deviations of the CO data are (1.66, 1.26), (0.49, 0.40), and (0.67, 0.25), respectively. Box plots of ambient temperature are shown in (**d**–**f**). Median temperature values are 17.8 °C, 29.3 °C, and 25.4 °C, respectively. Box plots of relative humidity (RH) are shown in (**g**–**i**). Median RH values are 48.9%, 28.5%, and 52.1%, respectively. All data are presented in hourly averaged samples.

Table 2 provides a summary of the three datasets collected from two locations in Italy and one in China. These datasets are multisensory, but we have focused on the calibration of the CO sensor as this gas is an essential component of the Air Quality Index (AQI) [55], and both the raw (from low-cost sensors) and reference CO data are available for all three deployments. We found that the data collected by the cost-effective multisensory devices and reference sensors have missing samples. Previous research has found evidence of cross-sensitivity in these gas measurements (e.g., see [21]). Therefore, for any given instant, all pollutant (and temperature and relative humidity) data need to be available from the cost-effective sensor alongside the reference CO data for multivariate calibration. As a result, we removed readings of select time instants from each dataset if any pollutant data from the cost-effective sensors or the CO ground truth data were missing. Please see Figure 1 for the CO ground truth distributions and temperature/relative humidity data for all three datasets. World Health Organization (WHO) recommended limits for CO exposure are no more than 9–10 ppm/8 h, 25–35 ppm/1 h, and 90–100 ppm/15 min [56]. As can be seen, the CO concentrations in all three monitoring sites are lower than these thresholds.

### 2.1. Dataset 1

The dataset was recorded by a multi-sensor device [37] containing an array of five low-cost MOX sensors that measured CO, NO_2_, O_3_, Non-methanic Hydrocarbons (NMHC), and NOX along with temperature (T) and relative humidity (RH). It includes 9357 samples of hourly averaged responses recorded between 10 March 2004, to 4 April 2005, from the Lombardy Region, Italy. A co-located certified reference analyzer provided ground truth, a conventional monitoring station with a spectrometer [37] that provided hourly averaged CO concentrations. After removing missing data points, we were left with 6941 samples for each pollutant, T, RH, and CO ground truth. More details of the dataset can be found in [17,21].

### 2.2. Dataset 2

This dataset includes the responses of a MONICA multi-sensor device [44] deployed in the Italian city of Naples. The gas sensor hardware consists of an array of electrochemical gas sensors to measure CO, NO_2_, and O_3_, along with T and RH. Hourly average responses were recorded along with reference CO concentrations from a certified analyzer (Teledyne 300, manufactured by Teledyne API). After discarding the missing data, a total of 13,595 samples collected over 31 months (5 April 2018–24 November 2020) are available. More details of the dataset can be found in [28]. It should be noted that the auxiliary electrode data of the CO data is available for the MONICA sensor and has been utilized during calibration.

### 2.3. Dataset 3

This dataset was recorded by a Sniffer4D multi-sensor device [29] deployed in the Chinese city of Guangzhou. The array of EC gas sensors measured CO, NO_2_, and O_3_ along with T and RH. A total of 3450 samples of hourly average data collected over a span of six months between 1 October 2018, and 1 March 2019, are utilized along with reference CO concentration collected from a certified analyzer (Thermo Scientific 48i-TLE). More details of the dataset can be found in [29]. Please note that this dataset is also available at a higher per-minute sampling rate.

## 3. Methodology

The calibration was framed as a supervised regression problem such that

(1) $${\mathrm{CO}}_{calibrated}=\Phi \left\{{\mathrm{CO}}_{raw},\underline{X}\right\}$$

Here ${\mathrm{CO}}_{calibrated}$ is the calibrated CO reading computed from the raw CO reading of the sensor (${\mathrm{CO}}_{raw}$) and $\underline{X}$, that comprises covariate factors, such as T, RH, and other pollutant readings from the sensor array (e.g., uncalibrated NO_2_ and O_3_ readings from the low-cost sensor array). For Dataset 2, ${\mathrm{CO}}_{raw}$ includes both the working electrode and auxiliary electrode data. Φ is the regression model whose parameters are derived from the training data to minimize the Mean Square Error (MSE) between the calibrated output and the ground truth received from the reference CO sensor. The training set is a subset of the dataset. We have considered two different Train Test Split (TTS) for this study. In TTS1, each of the three datasets is split so that 90% of the data is used to train (and validate, as discussed later) the calibration model, while the remaining 10% is used for evaluating the performance of the trained model. This 90/10 split represents the scenario where a co-located low-cost sensor is being used as a backup in case the reference grade monitor is out of commission for a short period due to fault or maintenance. In TTS2, the train/test split is 20/80. This emulates a scenario when a low-cost sensor is co-located with a reference sensor for a set period for calibration and afterward deployed in the field for monitoring pollutants at locations where no reference AQM station is available. It should be noted that for both the train test splits, we have used consecutive samples. The first 90 or 20 percent samples were used for training, and the remaining data were used for testing. This imitates a practical scenario where the sensor is collocated with the reference for a set period of time for calibration and then taken for field deployment. This also helps the calibration algorithms to exploit the temporal correlation between contiguous samples.

Three different regression cases were considered for each of the ML algorithms.

### 3.1. Scenarios 1–3

#### 3.1.1. Scenario 1 (SC1)

This involves deriving regressors or calibration models so that

(2) $$CO_{calibrated}^{SC1}=\Phi^{SC1}\left\{{\mathrm{CO}}_{raw}\right\}$$

The regressor, $\Phi^{SC1}$, is derived solely based on the raw CO sensor input to minimize the MSE between $CO_{calibrated}^{SC1}$ and the ground truth. For datasets 1 and 3, ${\mathrm{CO}}_{raw}$ represents the working electrode data of the low-cost CO sensor. For dataset 2, ${\mathrm{CO}}_{raw}$ comprises both working and auxiliary electrode data.

#### 3.1.2. Scenario 2 (SC2)

The second case introduces temperature and relative humidity readings as part of the input so that

(3) $$CO_{calibrated}^{SC2}=\Phi^{SC2}\left\{{\mathrm{CO}}_{raw},T,RH\right\}$$

The regressor, $\Phi^{SC2}$, is now derived from three input variables, raw CO sensor data, temperature, and relative humidity, to minimize the MSE between $CO_{calibrated}^{SC2}$ and the ground truth. Accurate T and RH sensors are inexpensive, and it is reasonable to expect the availability of these readings for any deployment. As mentioned before, the literature suggests that low-cost gas sensor operations are impacted by T and RH. Therefore, introducing a multivariate calibration strategy is the next logical step.

#### 3.1.3. Scenario 3 (SC3)

Cross-sensitivity is a known issue with low-cost gas sensors. However, this dependency can also be exploited to improve the calibration if the covariate pollutant data is available. In fact, it is quite common to construct and deploy a sensor array consisting of multiple pollutant sensors (as was the case in the three deployments that produced the datasets used in this research). Therefore, the last case further introduces other pollutant readings from the sensor array as part of the input that leads to

(4) $$CO_{calibrated}^{SC3}=\Phi^{SC3}\left\{CO_{raw},T,RH,NO_{2}^{raw},O_{3}^{raw}\right\}.$$

### 3.2. Machine Learning Algorithms

**Figure 2:**
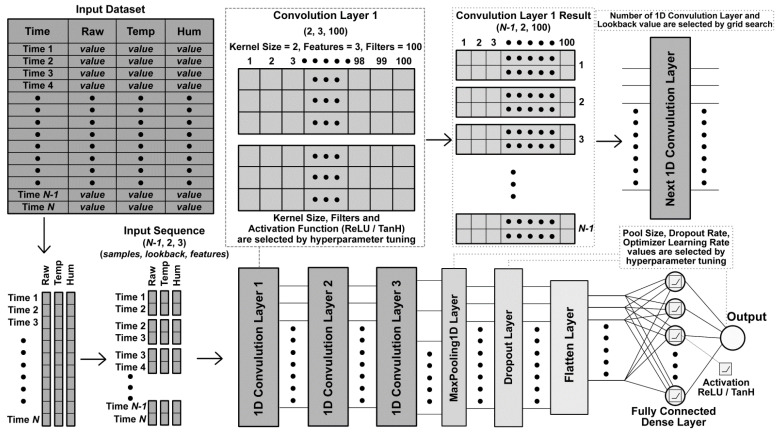
An example of a 1DCNN model developed for this study. Please note that many parameters are determined through grid search and tuning. Therefore, they vary depending on the data set, scenario (input variables used), and time split.

Convolutional Neural Networks (CNNs) have become a popular machine learning technique during the last decade [57]. Conventional CNNs are mainly designed to process two-dimensional (2D) data, e.g., videos and images [58]. This structure can be modified as 1DCNN to deal with one-dimensional signals [59,60,61]. The 1DCNN algorithms have less computational complexity, compact structure (1–2 hidden CNN layers), and are less time-consuming to train, and thus are suitable for low-cost real-time applications as compared to their 2D counterparts [58]. In this paper, we propose to use the 1DCNN-based regressor for the calibration of low-cost CO sensors. 1DCNN is well-suited to deal with time series. Figure 2 shows an example of the 1DCNN structure used in our work.

As mentioned in Section 1, we also develop calibration models using GBR, an ensemble learning technique that has not been widely utilized for gas sensor calibration.

#### ML Algorithms for Benchmarking

We trained and evaluated/benchmarked the calibration performance of 1DCNN and GBR alongside three other ML-based techniques commonly reported in the literature, as discussed in Section 1. These are

- MLP has been employed in many reported works on gas sensor calibration. Please note that in the literature, it is sometimes referred to as an Artificial Neural Network (ANN), Feedforward Neural Network (FNN), Back Propagation Neural Network (BPNN), or simply Neural Network.
- Recent literature suggests that Recurrent Neural Networks, or RNNs, are well suited for sensor calibration due to their ability to exploit temporal correlation in the data. After some preliminary investigation, we selected Long Short-Term Memory (LSTM) as the RNN-based technique for our benchmark work.
- Random Forest Regressor, or RFR, is an ensemble learning technique that has shown good performance in several works for low-cost gas sensor calibration and was, therefore, also selected to benchmark against.
- Furthermore, linear regression is the most commonly employed technique for calibrating low-cost gas sensors and is, therefore, also utilized for benchmarking purposes.

**Figure 3:**
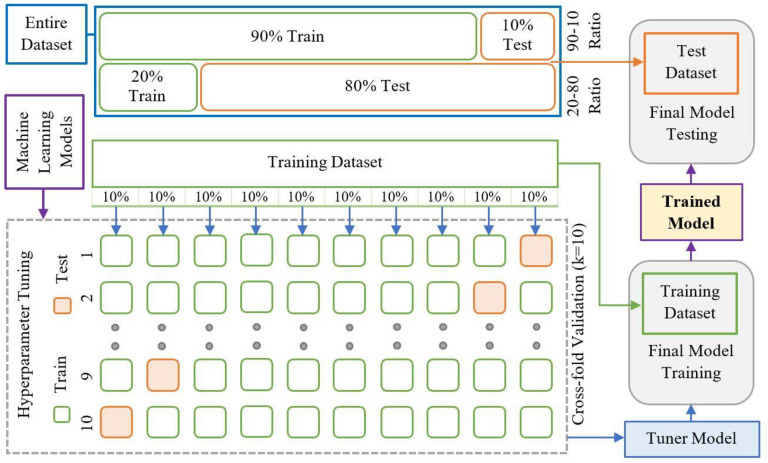
Details of hyperparameter training.

A rigorous training, validation, and testing approach has been followed in this work. All the regressors have hyperparameters that have been tuned on the relevant training datasets and tested on the corresponding testing sets. One way to make sure that the parameters are more generalized is through validation. A *k*-fold (*k* = 10) cross-validation has been implemented in this work. Multiple models with various hyperparameters are trained on the training dataset. The trained models are tested on the validation dataset, and the best-performing model is selected. The best-performing model is then finally trained using both the training and validation datasets. Lastly, this newly trained model is evaluated on the testing dataset. The following steps and Figure 3 provide a detailed description of this process:

Step 1: The dataset is split into training and testing datasets.

Step 2: The training is conducted using a ten-fold cross-validation, where the training dataset is divided into ten equal-sized parts. Each time nine out of the ten parts are used to perform a grid search for hyperparameters tuning and then evaluated against the remainder 10th part (validation). This process is repeated ten times, and the best hyperparameter combination is found across all ten evaluations.

Step 3: The best-performing model is further trained using the entirety of the training dataset. This training is done ten times over, and an average value of the predicted output is calculated.

Step 4: The final output is evaluated on the (unseen) testing dataset by computing the performance metrics.

Table 3 lists the hyperparameters that were tuned for all the ML algorithms. The final hyperparameters for every calibration model can be found online (https://github.com/Sharafat-Ali/AirQualityResults, accessed on 19 December 2022).

**Figure 4:**
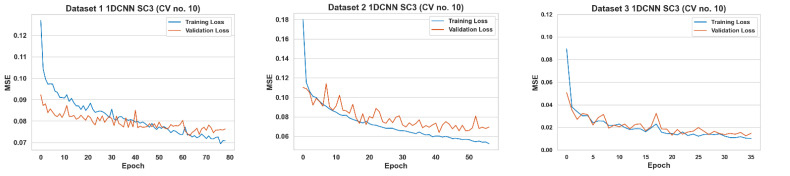
Example of training and validation losses for 1DCNN during k-fold cross-validation.

Instead of letting the training datasets run for a set number of epochs, an early stopping method has been used during the training-validating stage. Given the extensive and differing number of hyperparameters used across the various ML algorithms, it is difficult to exhaustively define the parameters in the manuscript. We have added a reference for interested readers [62]. This method allowed the training to end once the model performance stopped improving on the validation set. The validation sets’ mean absolute error (MSE) was monitored for each epoch. The training would stop when the MSE ceased to decrease by a certain tolerance threshold for a select number of epochs (patience). The model weights with the minimum MSE within that patience were taken as the validation set’s final weights. Figure 4 shows an example of the training and validation losses for 1DCNN.

Several performance metrics have been used in this study to benchmark and evaluate the different calibration models. These metrics, in various ways, measure the residuals or errors, i.e., deviations of the calibrated output of the low-cost sensors ($CO_{calibrated}$) from the ground truth ($CO_{reference}$) for the test data (10% or 80% of every dataset, depending on the split) that has never been used for training.

### 3.3. Performance Metrics

The Root Mean Square Error (RMSE), which is the standard deviation of the residuals and is commonly used as a performance metric for sensor calibration [29,63,64,65,66], was used where

(5) $$RMSE=\sqrt{\frac{1}{N}\overset{N-1}{\underset{i=0}{\sum }}{\left[CO_{calibrated}-CO_{reference}\right]}^{2}}$$

Here *N* is the number of samples in the relevant test data set.

Another metric utilized is the Coefficient of Determination ($R^{2}$), which is the goodness of fit in regression analysis [67,68,69]. It is computed as

(6) $$1-\frac{\sum_{i=0}^{N-1}{\left[CO_{calibrated}-CO_{reference}\right]}^{2}}{\sum_{i=0}^{N-1}{\left[CO_{reference}-mean\left(CO_{reference}\right)\right]}^{2}}$$

While the appropriateness of R^2^ to determine the fit of nonlinear regressors has been questioned, it is still commonly used within the discipline of air pollutant measurement (e.g., see [14,15,40,66,68]).

In some instances, we have also plotted the Cumulative Distribution Function (CDF) of absolute errors, $abs\left[CO_{calibrated}-CO_{reference}\right]$, for a more detailed investigation.

Target diagrams [15,40] were constructed to visualize the calibration models. The y-axis represents the Mean Bias Error (MBE) normalized by the standard deviation of the ground truth so that

(7) $$MBE=mean\left(CO_{calibrated}\right)-mean\left(CO_{reference}\right)$$

(8) $$Normalised\;MBE=\frac{MBE}{\sigma_{reference}}$$

Here, $\sigma_{reference}$ is the standard deviation of the ground truth for the relevant test data set. The *x*-axis of the diagram represents the normalized unbiased estimate of the RMSE, the Centered RMSE (CRMSE) given as

(9) $$CRMSE=\sqrt{RMSE^{2}-MBE^{2}}$$

(10) $$Normalised\;CRMSE=\frac{CRMSE}{\sigma_{reference}}$$

The normalized CRMSE is multiplied by $sign\left\{\sigma_{calibrated}-\sigma_{reference}\right\}$ to produce the target diagrams with $\sigma_{calibrated}$ being the standard deviation of the calibrated data for the relevant test data set.

## 4. Result & Discussion

Table 4 shows the performance of the calibration algorithms for all datasets in terms of RMSE (in ppm) and R^2^. We can make the following observations:

1. The accuracy of every calibration algorithm improves (lower RMSE, higher R^2^) as we go from SC1 (CO only) to SC2 (CO with T & RH) to SC3 (all inputs). The accuracy improves when temperature and humidity are included (SC2) alongside the raw CO data. There is further improvement in accuracy when the other pollutant data are also introduced (SC3) to exploit the dependencies arising from cross-sensitivity. This clearly emphasizes the importance of deploying low-cost sensors as multi-sensor platforms. Not only that allows for monitoring multiple pollutants with a single unit, but the accuracy of the measured data also improves.
2. Every ensemble and neural network-based algorithm outperforms linear regression-based calibration methods for all scenarios. In almost every instance, 1DCNN is the best-performing algorithm. This shows that 1DCNN could potentially improve the relative accuracy of low-cost multi-sensor air pollutant monitors. GBR and LSTM are the subsequent most accurate algorithms. While LSTM has gained much attraction for gas sensor calibration, GBR-based calibration appears to have received far less attention and warrants further investigation.
3. The accuracy of any given algorithm is better for the 90/10 split (TTS1) compared to the 20/80 split (TTS2). Interestingly, the accuracy improvement from SC1 (CO only) to SC3 (all inputs) is more noticeable than from TTS2 (20/80) to TTS1 (90/10). The covariate factors appear more important than longer training/co-location time. For example, consider the performance of 1DCNN for Dataset 1. The RMSE improves from 0.599 ppm to 0.393 ppm from SC1 (CO only) to SC3 (all inputs) for TTS2 (or from 0.542 ppm to 0.349 ppm for TTS1). Whereas for SC3 (all inputs) models, the RMSE improves from 0.393 ppm to 0.349 ppm when going from TTS2 (20/80) to TTS1 (90/10) (for SC1, it is from 0.599 ppm to 0.542 ppm).
4. The accuracy of the models derived and evaluated with TTS2 (20/80) is not significantly worse than those for TTS1 (90/10). This seems to suggest that with sophisticated calibration models, such as the ones presented in this work, not only the low-cost sensor platforms could be utilized as a backup for a reference grade monitor (TTS1), but they can also be deployed for reasonably accurate CO monitoring for a long duration after a short co-location (TTS2). It should be noted that the accuracy of the calibration models could be further improved by further periodic co-location and recalibration (please see [44]).
5. The accuracy of the algorithms appears to be the best for dataset 3. It could be due to the comparatively small number of low-concentration CO readings in dataset 3. The low-cost sensors typically struggle to register low gas concentrations. This is corroborated later in the section with box plots of residuals. Dataset 3 covers a considerably shorter time; therefore, the sensor may have experienced lower drift and degradation than the sensors of the other two measurement campaigns. It should be noted that the sensor platforms used to collect datasets 2 and 3 are constructed from the same CO sensors and therefore presents an opportunity for a reasonably objective evaluation of this effect.

**Figure 5:**
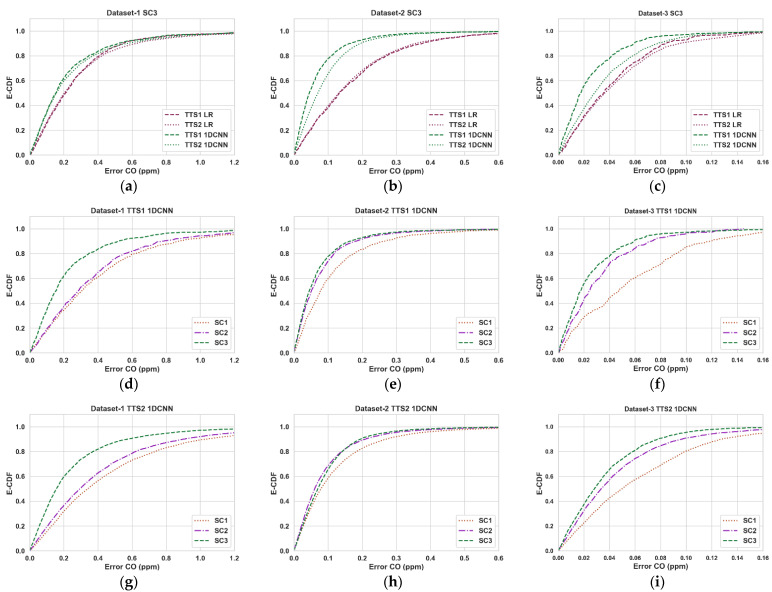
Empirical CDF plots of calibration error for 1DCNN. (**a**–**c**) Compare the CDF of error of the 1DCNN algorithm to the same of linear regression for both 90/10 (TTS1) and 20/80 (TTS2) splits for scenario 3 (with all available input parameters) for datasets 1–3. (**d**–**f**) Show how the CDF of er-ror of the 1DCNN algorithm improves from scenarios 1–3 for TTS1. (**g**–**i**) Show how the CDF of error of the 1DCNN algorithm improves from scenarios 1–3 for TTS2.

Figure 5 shows the CDF of absolute errors for the 1DCNN-based calibration (Error CDF plots for all scenarios can be found at https://github.com/Sharafat-Ali/AirQualityResults, accessed on 19 December 2022). The impact of T and RH on accuracy improvement seems more prominent for datasets 2 and 3. However, the cross-sensitivity impact to other pollutants appears more significant for Dataset 1. This could be because datasets 2 and 3 were collected using EC sensors, whereas those used for dataset 1 are MOX-based. It should be noted that these observations are consistent across all calibration techniques.

**Figure 6:**
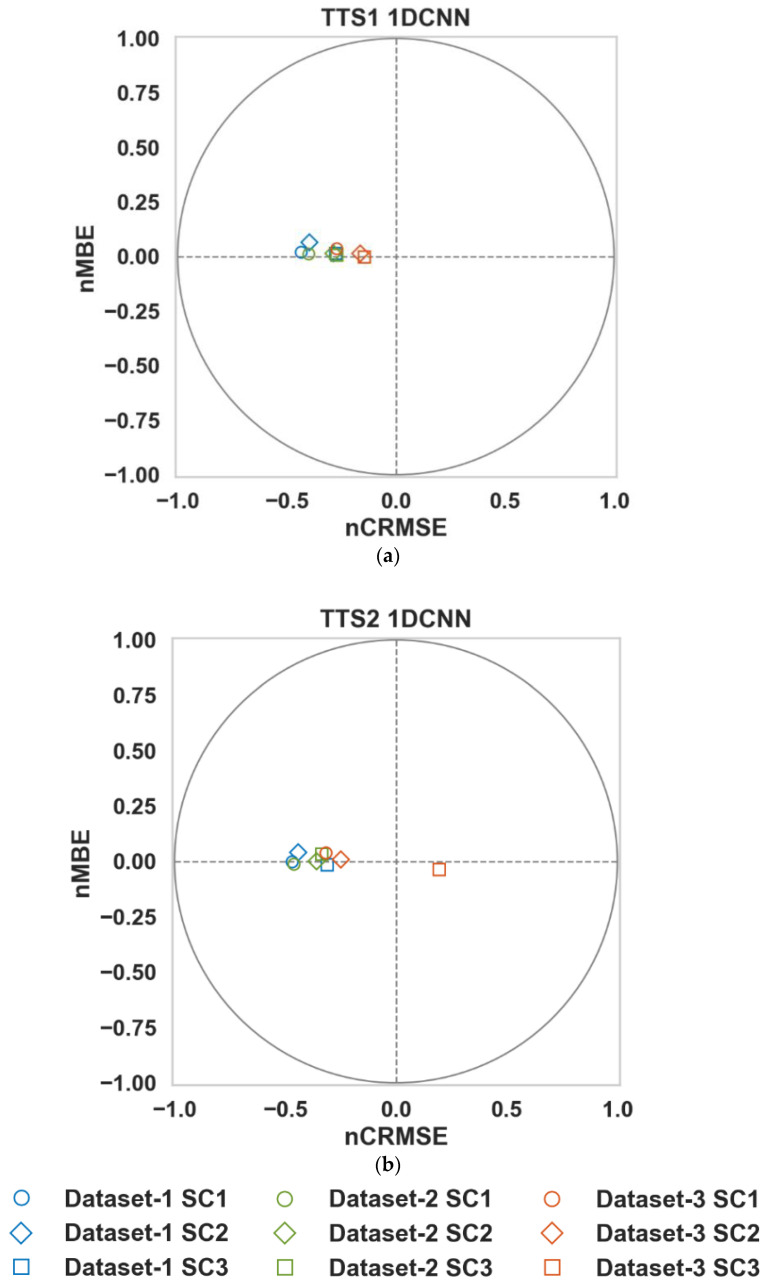
Target diagrams of 1DCNN for Datasets (**a**) TTS1 (90/10) and (**b**) TTS2 (20/80).

Target diagrams for the 1DCNN-based calibration models are shown in Figure 6 (Target diagrams for all algorithms can be found at https://github.com/Sharafat-Ali/AirQualityResults, accessed on 19 December 2022). The following observation can be made:

1. All points lie within the unit circle (radius = 1); therefore, the variance of the residuals is smaller than the variance of the reference measurements. It is an essential characteristic of a functional calibration model [15], indicating that the variability of the dependent variable (calibrated output) is explained by the independent variable (the reference data) and not the residual [27]. It should be noted that all calibration algorithms presented in this work fulfill this criterion.
2. The distance from the origin, which measures the normalized RMSE (RMSE/$\sigma_{reference}$) clearly show that the SC3 (all covariate inputs) regressors are more accurate than the SC2 (CO with T and RH) regressors, which are more accurate than the SC1 (CO only) regressors. This once again demonstrates the importance of the availability of covariate factors, such as temperature, relative humidity, and other pollutants.
3. The majority of the points lie on the left plane, indicating that the standard deviation of the calibrated sensor data for most models is smaller than the ground truth standard deviation.
4. For TTS1 (90/10), the points lie above the x-axis, indicating that the models, on average, slightly overestimate the CO concentration. For TTS2 (20/80), a few models also slightly underestimate the CO concentration.

**Figure 7:**
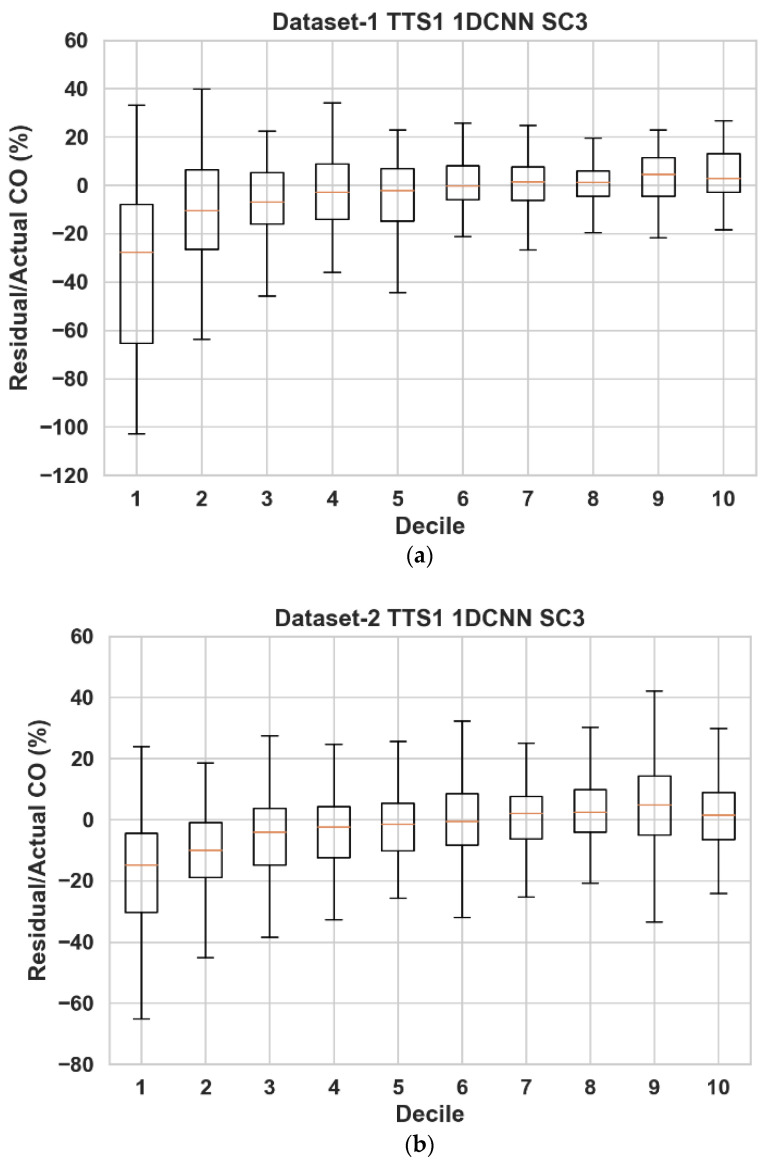

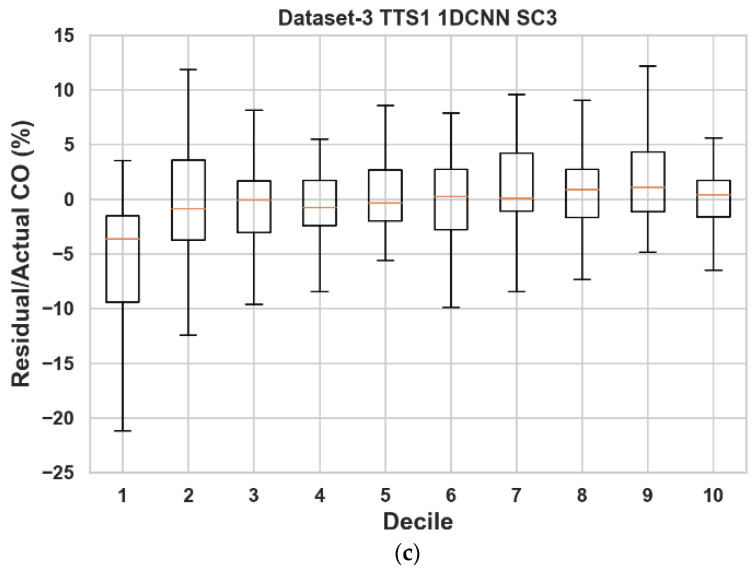
Box plots of residuals from the 1DCNN algorithm as a percentage of reference CO for TTS1 (90/10) with all available input parameters (SC3). (**a**) Datasets 1. (**b**) Datasets 2. (**c**) Datasets 3.

Low-cost sensors have been reported to suffer from variability in accuracy for different dynamic ranges of pollutants [27,40]. Therefore, we performed a quantitative investigation by constructing box plots of residuals as a percentage of ground truth at each decile of the ground truth (please see Figure 7 for the performance of the 1DCNN-based calibration). The figures show that at a lower concentration range of CO, the accuracy is the worst (median values further from zero) and exhibits more variability (larger boxes). The models underestimate at lower concentrations and slightly overestimate at higher concentrations. The variability appears to be more prominent for datasets 1 and 2. The likely reason for such increased variability and degraded performance is the difference in CO level experienced during the measurement campaigns. For datasets 1 and 2, the first deciles of CO data start at 0.0873 ppm and 0.066 ppm, respectively, compared to dataset 3 (starting at 0.296 ppm). Since the low-cost sensors are likely to struggle with sensitivity at low pollutant concentrations [22,70], this hardware limitation is probably causing performance degradation at the lowest deciles for datasets 1 and 2.

### Computational Cost

The computation cost of the ML algorithms can be mainly attributed to the training and tuning of the hyperparameters. However, it should be noted that these activities are not performed on sensor devices, which are constrained in terms of computational capability and energy storage. The low-cost sensors are deployed to collect and send the data to a backend server or a cloud-based infrastructure that performs the calibration/training offline. By investing in this infrastructure, we can deploy a city-wide low-cost sensor network at a very high spatial resolution. This is far more cost-effective than deploying a large number of expensive reference-grade sensors. We trained the ML algorithms on a workstation equipped with a 16-core AMD Ryzen processor, 128 GB RAM, and two NVIDIA A40 GPUs. The cost of this workstation (less than $20,000) is far lower than a single reference-grade gas sensor.

Once an algorithm has been trained, it can produce a calibrated output almost instantaneously. For example, even for the largest dataset (dataset 2), the trained 1DCNN algorithm takes less than 10 s to produce the calibrated output for the entirety of 80% of the data (20/80 split) for SC3, even on a simple PC (Intel Core i7-8700 3.20GHz CPU, 16 GB RAM) with no additional GPU. Therefore, it is possible to have a real-time operation with ML-based algorithms, where the data (electrode reading, T, RH, other pollutant readings) for one time instant will be sent to the cloud for the algorithm to produce the calibrated output only for that instant.

The computational complexity of ML algorithms can be benchmarked by comparing the total number of learnable parameters. Table 5 shows the number of learnable parameters for the three most consistent ML algorithms (1DCNN, GBR, and LSTM) and the linear regression for scenario 3 for the 90/10 splits (TTS1). The performance improvement of the ML algorithms comes at the cost of increased computational complexity, and GBR requires the highest number of parameters to be learned.

The number of learnable parameters increases as more covariate factors are included. Table 6 shows the total number of learnable parameters for 1DCNN for all three scenarios for the 90/10 splits. We can observe that the number of learnable parameters increases as we go from SC1 to SC2 and then from SC2 to SC3. Thus, the increased accuracy comes at the cost of learning more parameters.

## 5. Conclusions and Future Work

We proposed 1DCNN-based multivariate regressors for the calibration of low-cost CO sensors. The 1DCNN-based calibration algorithms were benchmarked against several machine learning-based calibration techniques and linear regression and were found to be the most accurate based on several performance metrics. GBR-based calibration models also, in general, perform better than many of the popular ML techniques for all datasets. Therefore, both 1DCNN and GBR should be further explored for low-cost gas sensor calibration.

We can also conclude that the CO sensors’ accuracy can be significantly improved by using data from other sensors of a multi-sensor platform. Therefore low-cost sensors should be deployed as multi-sensor arrays. It also appears that low-cost CO sensors can be reasonably accurately calibrated through a short co-location with a reference sensor and then deployed for a significantly more extended period for monitoring.

Data augmentation can improve the performance of ML algorithms and can be explored for improving the accuracy of the calibration algorithms. It should be noted that the sensors were tested on data that were collected from the same location. It is not apparent how the calibration models would perform for the same sensors located in an area with significantly different pollutant concentrations and meteorological conditions. In fact, the accuracy of ML algorithms may deteriorate when dealing with data that are outside of the calibration range. One way to deal with this issue would be to train multiple models at various concentration levels or distributions, and a heuristic-based algorithm can then be utilized to switch between the models depending on the pollutant concentration. The sensors will need to be collocated at multiple locations (e.g., central city, industrial area, suburb, etc.) to develop multiple calibration models. The models calibrated for a certain CO concentration can be used to develop the calibration models for other concentration levels. Future work can investigate the efficacy of such a strategy and transfer calibration if the same sensor-platform data is available from multiple locations.

In this work, we have only calibrated CO data. All three datasets also contain the raw and reference data for NO_2_. In the future, we will investigate and benchmark the performance of 1DCNN and GBR-based calibration for NO_2_.

Since low-cost sensor platforms are not likely to have an identical response, one might need a slightly different calibration model for each sensor. However, the development of such calibration models can be expedited through transfer calibration, where a base model, developed through extensive collocated deployments, is fine-tuned with a short collocation to address the slightly dissimilar hardware responses. This can be explored in a future study.

## Figures and Tables

**Table 1:** Summarization of the ML techniques used for the calibration of low-cost gas sensors.

ML Technique	Key Aspects	References
Multiple Linear Regression (MLR)	Classical statistical regression technique that uses a linear combination of several explanatory variables (e.g., temperature, relative humidity, etc.) to compute the calibrated sensor output.	[6,27,28,29,30,31,32,33]
Random Forest Regression (RFR)	An ensemble learning method for regression that develops a nonlinear regression model that uses several explanatory variables (e.g., temperature, relative humidity, etc.) to compute the calibrated sensor output.	[8,34,36,40,41,42,43]
Support Vector Regression (SVR)	A supervised learning algorithm that uses several explanatory variables (e.g., temperature, relative humidity, etc.) to compute the calibrated sensor output.	[34,35,36,37,38,39]
Multilayer Perceptron (MLP)	A classical neural network that uses backpropagation for training to develop a model that uses several explanatory variables (e.g., temperature, relative humidity, etc.) to compute the calibrated sensor output.	[25,27,28,37,38,39,43,44]
Recurrent Neural Networks (RNN)	A neural network that extracts data’s sequential characteristics and then uses backpropagation through time algorithm develops a model that uses several explanatory variables (e.g., temperature, relative humidity, etc.) to compute the calibrated sensor output.	[37,38,39,40,45,46]

**Table 2:** Details of the three datasets used in this study.

Dataset	Time Span (Days)	Location	Number of Samples	Low-Cost Sensor Array	Other Pollutant Measured	Reference CO Sensor
1 [37]	391	Lombardy Region, Italy	6941	MOX	NO_2_, O_3_, NMHC, NO_X_	Fixed conventional monitoring station equipped with spectrometer analyzers
2 [44]	965	Naples, Italy	13595	EC	NO_2_, O_3_	Teledyne T300
3 [29]	152	Guangzhou, China	3639	EC	NO_2_, O_3_	Thermo Scientific 48i-TLE

**Table 3:** List of hyperparameters that were tuned for each ML-based algorithm.

Algorithm	List of Hyperparameters
RFR	Maximum depth of the tree, maximum number of leaf nodes, and number of trees in the forest.
GBR	Maximum depth of the individual regression estimators, minimum number of samples required to be at a leaf node, minimum number of samples required to split an internal node.
MLP	Number of hidden layers, number of neurons in the hidden layer, activation function in the hidden layer, dropout rate in the dropout layer, the learning rate of the optimizer, and batch size.
LSTM	Number of LSTM layers, time steps, number of units in the LSTM layers, activation function, the dropout rate in dropout layers, the learning rate of the optimizer, and batch size.
1DCNN	Number of 1D convolution layers, lookback, number of filters in the convolution layer, activation function in the convolution layer, kernel size, pool size in max pooling layer, the dropout rate in the dropout layer, number of neurons in the dense layer, activation function in the dense layer, the learning rate of the optimizer, batch size.

**Table 4:** The accuracy of the calibration algorithms in terms of R^2^ and RMSE (in ppm). The best performance for each scenario is bolded.

Dataset	Test Train Split	Scenario	Performance Metric	Algorithm					
				LR/MLR	RFR	GBR	MLP	LSTM	1DCNN
Dataset 1	TTS1	SC1	RMSE	0.554	0.554	0.556	0.551	0.545	**0.541**
		SC2		0.543	0.508	0.506	0.526	**0.501**	0.502
		SC3		0.384	0.346	0.349	0.350	**0.344**	**0.344**
	TTS2	SC1		0.613	0.609	0.609	0.613	0.600	**0.598**
		SC2		0.597	0.594	0.594	0.588	0.572	**0.564**
		SC3		0.437	0.404	0.409	0.415	0.405	**0.396**
	TTS1	SC1	R^2^	0.803	0.806	0.805	0.810	0.808	**0.811**
		SC2		0.812	0.837	0.838	0.833	0.838	**0.841**
		SC3		0.905	0.924	0.923	0.923	**0.925**	0.924
	TTS2	SC1		0.767	0.770	0.771	0.768	0.780	**0.781**
		SC2		0.779	0.781	0.781	0.786	0.798	**0.805**
		SC3		0.882	0.899	0.897	0.896	0.900	**0.904**
Dataset 2	TTS1	SC1	RMSE	0.305	0.175	**0.172**	0.252	0.175	0.173
		SC2		0.254	0.132	0.127	0.219	0.130	**0.124**
		SC3		0.234	0.129	0.120	0.204	0.119	**0.117**
	TTS2	SC1		0.300	0.187	**0.182**	0.238	0.183	0.185
		SC2		0.249	0.147	**0.145**	0.217	0.147	**0.145**
		SC3		0.229	0.143	0.141	0.188	**0.134**	0.136
	TTS1	SC1	R^2^	0.507	0.837	**0.842**	0.713	0.837	0.841
		SC2		0.658	0.907	0.914	0.755	0.909	**0.918**
		SC3		0.710	0.910	0.923	0.868	0.924	**0.927**
	TTS2	SC1		0.443	0.784	**0.795**	0.675	0.792	0.789
		SC2		0.616	0.865	0.869	0.713	0.870	**0.871**
		SC3		0.674	0.873	0.877	0.842	**0.890**	0.887
Dataset 3	TTS1	SC1	RMSE	0.075	0.075	**0.072**	0.073	0.074	**0.072**
		SC2		0.060	0.046	**0.044**	0.049	0.045	**0.044**
		SC3		0.054	0.043	**0.038**	0.049	0.039	**0.038**
	TTS2	SC1		0.080	0.082	0.082	0.082	0.080	**0.079**
		SC2		0.067	0.064	0.063	0.063	**0.062**	**0.062**
		SC3		0.060	0.060	0.053	0.056	0.053	**0.049**
	TTS1	SC1	R^2^	0.920	0.919	**0.926**	**0.926**	0.922	**0.926**
		SC2		0.948	0.970	0.972	0.965	0.971	**0.973**
		SC3		0.958	0.974	**0.980**	0.971	0.978	0.979
	TTS2	SC1		0.895	0.890	0.890	0.897	0.894	**0.901**
		SC2		0.927	0.933	0.936	0.936	**0.937**	**0.937**
		SC3		0.941	0.941	0.954	0.957	0.954	**0.967**

**Table 5:** The number of learnable parameters for GBR, LSTM, and 1DCNN in SC3 for TTS1.

Dataset	Algorithm	Number of Learnable Parameters
Dataset 1	LR/MLR	8
	GBR	1,750,000
	LSTM	35,371
	1DCNN	227,241
Dataset 2	LR/MLR	9
	GBR	22,500,000
	LSTM	137,021
	1DCNN	393,551
Dataset 3	LR/MLR	9
	GBR	14,400,000
	LSTM	287,701
	1DCNN	520,591

**Table 6:** The number of learnable parameters for 1DCNN in different scenarios.

Dataset	Scenario	Number of Learnable Parameters
Dataset 1	SC1	48,191
	SC2	58,921
	SC3	227,241
Dataset 2	SC1	73,241
	SC2	129,681
	SC3	393,551
Dataset 3	SC1	187,841
	SC2	400,601
	SC3	520,591

## Data Availability

Dataset 1 is open access and can be found here: https://archive.ics.uci.edu/mL/datasets/Air+Quality#, accessed on 19 December 2022. Datasets 2 and 3 were collected from De Vito et al. and Liang et al., respectively.
